# Neurogenic Thoracic Outlet Syndrome with Supraclavicular Release: Long-Term Outcome without Rib Resection

**DOI:** 10.3390/diagnostics11030450

**Published:** 2021-03-05

**Authors:** Niina Ruopsa, Leena Ristolainen, Martti Vastamäki, Heidi Vastamäki

**Affiliations:** 1 Research Institute Orton and Orton Orthopaedic Hospital, 00280 Helsinki, Finland; leena.ristolainen@orton.fi (L.R.); martti.vastamaki@orton.fi (M.V.); heidi.vastamaki@fimnet.fi (H.V.); 2 Department of Hand Surgery, Tampere University Hospital, 33521 Tampere, Finland; 3 Mehiläinen Neo Sports Hospital, 20520 Turku, Finland

**Keywords:** long-term results, operative treatment, scalenotomy, thoracic outlet syndrome, TOS

## Abstract

Our aim was to define clinical long-term outcome of surgery for neurogenic thoracic outlet syndrome without rib resection, and to find factors predicting long-term results. For the 94 patients, the main outcomes were pain, numbness, weakness, and upper-extremity function. The Quick Disabilities of the Arm, Shoulder and Hand (QuickDASH) survey, the Cervical-Brachial Symptom Questionnaire (CBSQ), and a numerical rating system served as functional outcome measures. Mean follow-up was 12.9 years. Preoperative pain diminished from 7.8 to 2.2, numbness from 7.4 to 4.0, and weakness from 7.3 to 3.8. Grip strength increased from 25.7 to 31.8 kg. QuickDASH averaged at follow-up 37.1 and CBSQ 51.5. No correlation appeared between smoking and long-term results regarding pain, numbness, weakness, or functioning. Positive TOS provocative tests or intraoperative anatomical findings like consistency of the scaleni muscles showed no correlation with outcome. 82% of female and 57% of male patients reported that aid from this surgery had been excellent or good; 69% reported that surgery helped considerably for at least a mean 9.9 years. The risk for worse self-reported long-term outcome was higher among men, but neither BMI nor age at surgery associated with self-reported outcome. Pain, numbness, and weakness significantly decreased and function improved after supraclavicular release without rib resection. We found no significant preoperative nor per-operative factors predicting long-term results.

## 1. Introduction

Neurogenic thoracic outlet syndrome (NTOS) is a disorder with diverse pathology including a range of symptoms related to the neck, shoulder, upper chest, head, and upper limb. Patients may present with pain, weakness, numbness, and paresthesia. The diagnosis can be demanding, mainly based on patients’ history and physical examination including provocative tests. X-ray, ultrasound, CT, MRI, angiography, and ENMG are additional tools in differential diagnosis [1].

Physiotherapy is the treatment of choice in patients with NTOS and often is beneficial, especially for its mild forms [2]. Vanti et al. [3] concluded that conservative treatment such as exercise, massage, cold therapy, and posture correction may prove effective in terms of pain reduction, return to work, and functional improvement. For patients who do not benefit from physiotherapy, surgical treatment is one option [4,5], in which three surgical methods are possible: scalenotomy or scalenectomy, first-rib resection, and scalenotomy plus first-rib resection. Reports on the long-term results of different methods are especially rare. Povlsen et al. [6] found that evidence of transaxillary first rib resection as reducing pain more effectively than supraclavicular neuroplasty was of very low quality. Peek et al. [7] found that long term functional outcome after first rib resection for TOS was satisfactory, but the mean QuickDASH (The Quick Disabilities of the Arm, Shoulder and Hand) scores remain higher than those of the general population. No randomized evidence exists, however, that either procedure is better than no treatment. In particular, reports on prediction of preoperative or per-operative signs are almost lacking.

Among surgeons, the topic of surgical NTOS management has been controversial, with an absence of objective diagnostic as well as of outcome criteria. Selection of NTOS patients who will truly benefit from surgical management has been difficult. Lack of clinical tests for NTOS to serve as predictors of outcome is also a problem. Ghamari et al. [8] used, in these patients, five generic outcome measurements to determine intervention outcomes. Clinical tests currently used show good sensitivity but poor specificity and cannot predict the outcome reliably [9]. The new Thoracic Outlet Syndrome Index (TOSI) may prove useful for outcome measurement [10].

Although short-term results show resolution of NTOS symptoms in as high a proportion as 90% of patients [11], long-term series show a gradual deterioration over time to as low as 50% to 60% [12,13]. There remains a lack of consensus as to the most appropriate surgical approach in NTOS management, as highlighted by Povlsen et al. [14], although many articles have compared the results of various operative methods [15,16,17,18,19]. Objective critical data on the success of such treatment are conspicuously lacking [2].

Our aim was to report clinical long-term outcome and discover factors predicting responsiveness to surgical treatment for neurogenic thoracic outlet syndrome without rib resection. We hypothesized that surgical treatment alleviates NTOS symptoms substantially and improves patients’ quality of life (QoL). We also felt optimistic concerning the discovery of preoperative and per-operative factors significantly predicting long-term results.

## 2. Materials and Methods

### 2.1. Patient Selection

**Figure 1:**
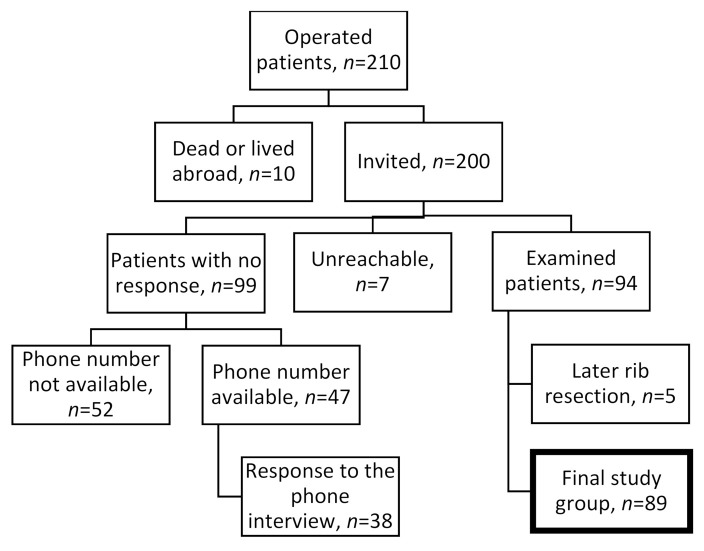
Flow chart of the operated NTOS patients.

We retrospectively performed a study on patients with neurogenic-type TOS who underwent surgery by a single surgeon in our facility. The study population comprised patients referred to a tertiary referral orthopedic hospital for evaluation and surgical treatment of NTOS. Patients excluded had arterial or venous forms of TOS in the absence of disabling NTOS. We considered that severe consistent paleness during a Roos test might reliably indicate tightness between the clavicle and first rib structures. Those patients, fewer than 10, we did not operate on but referred to thoracic surgeons for a first-rib resection. A total of 210 scalenotomy operations took place between 1997 and 2011. Of the 210 patients, 9 were deceased, and one lived abroad. Of the 200 patients invited, 7 were unreachable and 94 participated (47%). Of the 94, 5 patients (5.3%) had an unsatisfactory result and they had later a rib resection. All of these five patients were women. In addition, we contacted 47 others by phone (Figure 1).

The final study group, of the total of 141 contacted, thus comprised 89 (63%) with 13 (15%) having been operated on bilaterally. Of those 89 patients examined, 83% were female, but among non-respondents, 75% were female (*p* = 0.672). Respondents’ age was a mean 48.5 years, non-respondents’ 51.2 (*p* = 0.085) (Table 1).

### 2.2. Workup and Surgical Intervention

An important part of the workup for NTOS patients consisted of ruling out other pathology that could cause similar symptoms. A detailed history for each patient came from a prospectively maintained database and was summarized from office notes, hospital records, imaging studies, operative findings, and records from treating physicians and therapists. Clinical post-operative follow-up covered all cases. Before surgery, 86.4% of patients had undergone physiotherapy sessions a mean 26 times as conservative treatment. In 12 patients, the symptoms of TOS were so severe that physical therapy was too painful. We operated on these 12 without further experimentation with conservative treatment. As a rule, we try conservative treatment continuing for at least 3, preferably 6 months, before surgery. We did not use any scalene injections before the surgery.

Initial evaluation included the history and physical examination relevant to NTOS and review of previous evaluations, imaging studies, and electrophysiologic tests. The diagnosis of NTOS was predominantly by clinical criteria, as previously described [20]. Plain cervical spine radiographs allowed identification of bony abnormalities. In appropriate cases, a magnetic resonance imaging of the neck ruled out ruptured disk, spinal stenosis, or other abnormalities. Electrophysiologic testing served to exclude peripheral neuropathies or cervical nerve root syndromes as well as any true neurogenic TOS.

**Figure 2:**
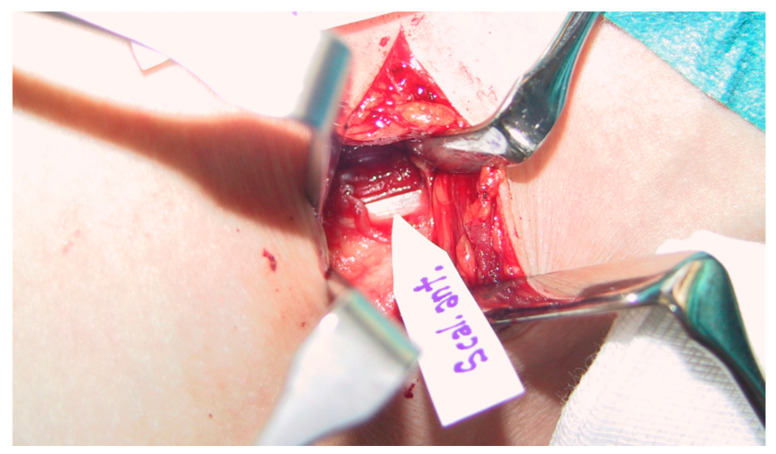
Intraoperative picture showing fascia of the scalenus anterior muscle.

**Figure 3:**
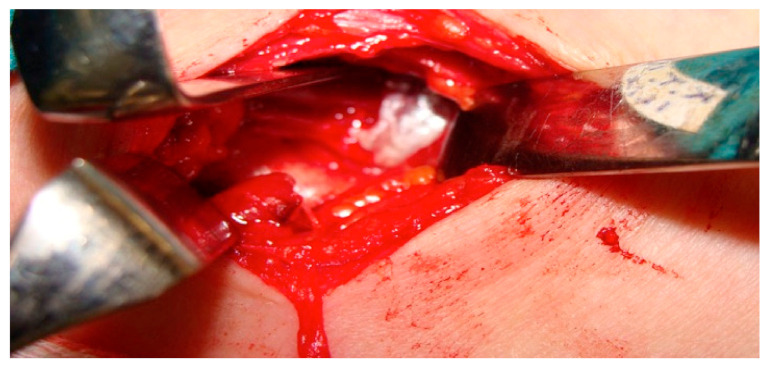
Intraoperative picture after discision of the scalenus anticus muscle showing the subclavian artery now free.

All patients were preoperatively examined and diagnosed as well as operated on by a surgeon specialized both in orthopedic and hand surgery and with long experience in TOS surgery (some 500 TOS surgeries). Scalenotomy was performed in the manner of Sanders et al. [17]. After dissection of the anterior scalene muscle, all connective tissue fibers, stray muscle fibers, and any another bands and ligaments were excised to completely expose the nerve roots with no drain used (Figure 2 and Figure 3).

Patients usually left the hospital one day post-operatively. Immobilization was unnecessary. Physiotherapy was on offer only exceptionally to those patients who failed to recover smoothly.

### 2.3. Long-Term Outcome

At the final follow-up, all patients completed the QuickDASH survey questionnaire [21], the CBSQ (the Cervical-Brachial Symptom Questionnaire) [22], and the 11-point numeric rating scale (NRS) for pain [23], and the study questionnaire. The QuickDASH is an 11-item survey scored on a 0–5 scale designed and validated for use in a variety of upper extremity musculoskeletal disorders to quantify degree of disability. The CBSQ is a 14-item survey scored on a 0–120 scale, developed for evaluation of patients with NTOS and related disorders to measure functional disturbances resulting from performance of certain activities. The NRS is a widely used standard method for assessment of pain.

The study questionnaire included the following: self-reported estimation of symptoms, anatomical areas involving these symptoms, and limitations caused by TOS before surgery and at final follow-up at a mean 12.9 years (5.9–21.6) after surgery. In addition, we recorded patients’ working status, use of physiotherapy, and estimation of how much and for how long the operation had improved their lives.

The final follow-up by two independent surgeons specialized in hand surgery included a clinical examination and grip and key pinch strength measurement for each hand. Tests were the Adson (in 90 degree abduction, head turned to the opposite side) and Roos (until one minute) as well as estimated range of movement (ROM) of shoulders. Supraclavicular tenderness, Tinel sign, and supraclavicular compression tests of the brachial plexus were other examinations. The scar was inspected, and length of scar measured. During the inclusion period, 10 patients underwent non-simultaneous bilateral procedures. The mean follow-up time was 12.9 years, and average duration of symptoms before TOS diagnosis was 4.4 years. No difference emerged in duration of symptoms by gender.

### 2.4. Statistical Analysis

Statistical data analysis included IBM SPSS Statistics (v. 26.0) using appropriate statistical tests such as frequencies, chi-square test, and t-tests. The paired sample t-test served for testing pain, numbness, weakness, daily harm before the operation and during the questionnaire period. One-way ANOVA was determined between variables such as results of the Roos elevated test and plexus tenderness at surgery, and pain during the questionnaire period. Logistic regression explained the relationship between pre-and intraoperative variables and long-term outcomes of surgery (Odds Ratios (OR) and its 95% Confidence Intervals (95% CI). Data were considered statistically significant at *p* < 0.05, two-tailed.

## 3. Results

### 3.1. Patient Characteristics

We identified a total of 94 patients with NTOS operated on by scalenotomy without first-rib resection (Figure 1). Of the 94, 5 patients (5.3%) had an unsatisfactory result and they had later a rib resection leaving 89 patients for analysis. Self-reported NRS pain diminished by 3.2, numbness by 3.4, and weakness 3.5 (each, *p* < 0.001) (Table 1). Of the patients, 63 (81%) reported daily pain before surgery, but at follow-up only 18% (*p* < 0.001). Before TOS surgery, 57% reported headache at least once a week, at follow-up only 30% (*p* = 0.021). Before surgery, 57% experienced every week a so called “deadly numb arm” symptom, at follow-up only 16% (*p* < 0.001). Before surgery, 94% reported major difficulties in hand-upward positions, at follow-up only 34% (*p* < 0.001). Grip strength in the operated hand averaged 26.2 kg (SD 11.6) before surgery and at follow-up 32.2 kg (SD 15.1) (*p* < 0.001), but key pinch, at 6.6 kg (SD 2.5), stayed the same.

### 3.2. Functional Outcome

At follow up, QuickDASH averaged 37.1 and CBSQ 51.5. TOS-related NRS daily harm diminished by 4.0 (*p* < 0.001) (Table 2).

Having a tendinous scalenus anterior muscle versus a normal or thick scalenus muscle seemed to be associated with daily harm at follow-up (OR 2.9, 95% CI 1.1–8.0, *p* = 0.038). More than half the women (54%) were still working full time, and 12% had a part-time job, 8% were unemployed, and 18% were retired, two because of NTOS symptoms. Of the men, 79% were still working full time, 7% unemployed, and 14% retired do to NTOS.

### 3.3. Subjective Outcome

Concerning patient’s subjective opinions, 87% of female and 57% of male patients reported that the result of surgery was excellent or good, and 82% would choose the same surgery again. Surgery helped 65 (73%) very much for at least a mean 9.9 (SD 6.1) years. The risk for worse self-reported long-term outcome was higher among men (OR 4.4, 95% CI 1.4–14.2), but neither BMI (OR 1.0, 95% CI 0.9–1.1) nor age at surgery (OR 1.0, 95 CI% 0.97–1.1) was associated with self-reported outcome. No correlation appeared between smoking and long-term results. Neither a positive Tinel sign, plexus compression sign, Adson or Roos’ elevated arm test, nor intraoperative anatomical findings such as consistency of the scaleni muscles showed any correlation with outcome (Table 3 and Table 4).

### 3.4. Complications

Physician-reported complications appeared in five patients: a transient phrenic nerve palsy, levator scapulae muscle pain for 4-5 days, dissection of the ventriculoperitoneal shunt inserted 30 years earlier, edema around the neck which caused problems in head movement, and a temporary serratus palsy. There were also 13 self-reported complications such as problems with subcutaneous stitches which had not been absorbed. Three patients had problems with breathing after surgery, but no permanent phrenic nerve or serratus palsies emerged.

### 3.5. Non-Participating and Excluded Patients

Of those non-responding, 47 (47.5%) could be contacted by phone; 16 had been too busy to take part in the research, 20 had unrelated disease hindering their participation, and others had reasons like too lengthy a trip to follow-up. Of those 47 interviewed, 59% judged their long-term result as being excellent or good. Five female patients were failures due to a later rib resection. Their outcome results after rib resection were similar to outcomes of others (Table 5).

## 4. Discussion

In NTOS patients after supraclavicular release without rib resection, we found that pain, numbness, weakness, and daily harm were significantly decreased. However, we found no preoperative or intraoperative factors significantly predicting the long-term results.

Sanders and Pearce [18] compared first-rib resection to scalenectomy. Both had similar results, with success of the latter surgery being 91% to 93% at 3 months; 76% to 79% at 1 to 2 years; 70% to 73% at 3 to 5 years; and 69% to 72% at 5 to 10 years. In our research, 74% of the patients reported that the result of scalenotomy was excellent or good in long-term follow-up of 12 years. Thus, rib resection seemed to be unnecessary in most of our NTOS patients.

Perchoc et al. [24] reported that after surgical treatment of TOS, pain was completely absent in 29% of cases and was reduced in 51%. In our study, 81% of the patients reported daily pain before surgery, but at follow-up only 18%. Weigel et al. [25] reported NRS pain improvement of 5.0, whereas our self-reported pain improvement was 3.8. Weigel’s study group was, however, quite small: only 10 patients. Of our patients, 66% reported that all or over half their pain symptoms disappeared with surgery. Sivertsen et al. [26] reported similar results. Zhang et al. [27] also found that after scalenus anticus resection, occipital headaches were completely relieved in 81% and partially relieved in 13%. In our series, 57% reported headache at least once a week before TOS surgery, and at follow-up only 30%.

Among our patients, 66% reported that after surgery all or over half their numbness disappeared. Grip strength improved significantly, as well. Gong et al. [28] found that paresthesia diminished significantly after TOS surgery, and Perchoc et al. [24] found TOS surgery to reduce the number of patients suffering from paresthesia, numbness, or weakness. The so-called dead-arm syndrome also was less often evident after surgery, but not significantly. Of our patients, 56% experienced every week the dead-arm syndrome preoperatively, but at follow-up only 16% did so (*p* < 0.001).

For us, that QuickDASH averaged, at follow-up, 37, meant that the patients had mild or moderate difficulties with most of their activities. That is at the same level as earlier [25,29,30]. Ransom et al. [31] reported a mean postoperative score of 11.4 for QuickDASH, but their patients’ being adolescents may explain this differing result. CBSQ at our follow-up averaged 52, at the same level as in Caputo’s research [29] but higher than for Glynn et al. [30], Ransom et al. [31] and Rochlin et al. [32].

Rochlin et al. [32] stated that patient factors, particularly comorbidities and opioid use, are more predictive of poor long-term QoL after TOS surgery than is preoperative scalene block. According to them, variables predictive of worse scores being an active smoker, being age ≥40 years, with chronic pain syndrome, general comorbidity, preoperative and postoperative use of opioids, and any surgical complications. We found no predictors. In our experience, men perhaps more often needed first-rib resection and their risk for worse self-reported long-term outcome was 4.4 times higher. In our opinion, intraoperative compression between the first rib and the clavicle was more often found in men, meaning that a first-rib resection was indicated. We have considered that severe consistent paleness during the Roos’ test might mean considerable tightness between these structures. However, our statistics did not show this. Those patients with such Roos’ test paleness we have referred for rib resection to thoracic surgeons during the last 20 years although we recognize that Roos’ test paleness should not be considered as a contraindication for scalenotomy alone.

Scalene muscle or pectoralis minor muscle blocks were not used in our study to confirm the diagnosis. The role of pectoralis minor should always be considered if tenderness under the pectoralis minor muscle is marked [33].

Ohman et al. [34] found no substantive influence of BMI on preoperative characteristics or intraoperative, postoperative, or 3-month outcomes for patients with NTOS after supraclavicular decompression. They also found no indication of an “obesity paradox” for this condition. Our results were similar. In Caputo’s series [28], adolescent patients improved better than did adults over age 21. We found no statistically significant differences between adolescents and adults, but we studied only seven adolescents. This study has several strengths. First, the patients were examined both with lengthy questionnaires and objectively clinically by experienced medical doctors specialized in hand surgery. Second, the follow-up time was long. Third, all patients were examined at first consultation and diagnosed and operated on by an orthopedic and hand surgeon experienced in TOS surgery. Only patients with appropriate status recordings were included. Fourth, the operations were mainly single-surgeon procedures. Fifth, the population in this study is large. We also used validated QoL instruments and qualitative patient assessment of symptom relief.

We acknowledge some limitations. First, we used no validated scores like QuickDASH or CBSQ preoperatively. Recall bias may be likely, when patients had to complete questionnaires concerning their preoperative situation at follow-up. Second, this retrospective follow-up case study lacks any control group. Third, some patients also had new symptoms, for instance shoulder pain, and these found it difficult to separate which of the remaining symptoms were caused by TOS and which by some new disorder. 5 patients had later a rib resection, but we do not have data of their situation after scalenectomy. Moreover, the dropout was large, as only 47% responded to our invitation. We interviewed 47% of the dropout group by phone but were unable to perform a clinical examination. Some differences may exist in results between patients who participated and those who did not.

At the time of our research, we had no specific thoracic outlet syndrome index, so the results of QoL were difficult to compare with those of earlier research. Vastamäki et al. [10] have now created a thoracic outlet syndrome index (TOSI). We recommend further studies with a randomized control trial using that index.

## 5. Conclusions

In conclusion, pain, numbness, weakness, and daily harm significantly decreased after supraclavicular release without rib resection in patients with disputed neurogenic thoracic outlet syndrome. Rib resection seems to be unnecessary in most NTOS patients. However, having still no significant preoperative or intraoperative factors predicting surgery outcome, means that we recommend further study.

## Figures and Tables

**Table 1:** Demographics of TOS-patients treated surgically with scalentomy without five failures with later first rib resection.

	Female	Male	All	*p*-Value
Sex, *n* (%)	74 (83)	15 (17)	89	<0.001
BMI at surgery time (kg/m^2^), mean (SD)	25.1 (4.3)	26.3 (3.7)	25.3 (4.2)	0.307
BMI at final follow-up (kg/m^2^), mean (SD)	27.2 (5.9)	26.7 (2.8)	27.1 (5.5)	0.720
Age at surgery (years), mean (SD)	35.6 (10)	35.7 (12)	35.6 (11)	0.997
Age at final follow up (years), mean (SD)	48.5 (11)	48.8 (13)	48.5 (11)	0.911
Follow-up (years), mean (SD)	12.8 (4.5)	13.2 (4.2)	12.9 (4.4)	0.784
Smokers at follow-up (%), *n* (%)	25 (36)	5 (33)	30 (35)	0.861
Right side affected, *n* (%)	47 (64) *	11 (73) **	58 (63)	0.454
Duration of symptoms before TOS-diagnosis (years), mean (SD)	4.4 (3.8)	5.3 (4.4)	4.4 (3.8)	0.397

*p*-value is distributed between female and male patients; * The number includes females with both hand operated (40 + 7); ** The number includes males with both hand operated (8 + 3).

**Table 2:** Long-term outcome after supraclavicular TOS surgery without first rib resection.

Assessment	Female Mean (SD) *	Male Mean (SD) *	All	*p*-Value
QuickDASH score (scale 0–100)				
At follow-up	39 (25)	31 (21)	37 (24)	0.326 **
CBSQ score (scale 0–120)				
At follow-up	51 (35)	52 (35)	52 (35)	0.927 **
Pain (NRS scale 0–10)				<0.001 ***
Preoperatively	8.0 (1.6)	6.7 (2.8)	7.8 (1.9)	0.122 **
At follow-up	4.0 (3.2)	3.8 (3.4)	4.0 (3.2)	0.799 **
Numbness (NRS scale 0–10)				<0.001 ***
Preoperatively	7.5 (1.9)	7.0 (2.5)	7.4 (2.0)	0.388 **
At follow-up	3.9 (3.0)	4.5 (3.1)	4.0 (3.0)	0.518 **
Weakness (NRS scale 0–10)				<0.001 ***
Preoperatively	7.4 (1.9)	7.1 (2.2)	7.3 (2.0)	0.578 **
At follow-up	3.7 (2.9)	4.3 (3.1)	3.8 (3.0)	0.517 **
Daily harm caused by TOS (scale 0–10)				<0.001 ***
Preoperatively	8.2 (1.4)	7.1 (2.0)	8.1 (1.6)	0.056 **
At follow-up	4.0 (3.1)	4.4 (3.2)	4.1 (3.1)	0.695 **
Result excellent or good, *n* (%)	54 (79 %)	7 (47%)	61 (74 %)	0.009 **

*CBSQ,* Cervical-Brachial Symptom Questionnaire; *QuickDASH*, Quick Disabilities of Arm, Shoulder and Hand; NRS, Number Rating Scale. * = Data shown represent the standard deviation (SD). *p*-value ** = Comparison between sex. *p*-value ***—paired sample t-test in patients, differences between before operation and during follow-up questionnaire time.

**Table 3:** Preoperative clinical findings of patients with TOS and the outcome.

Plexus Tenderness Roos’ Elevated Arm Test, 1 Min	Number	%	Outcome Excellent or Good %
No	22	28	68
Slight	27	35	67
Moderate	11	14	64
Severe	18	23	72
No Paleness	60	75	72
Slight Paleness In The Beginning	8	10	63
Temporary Paleness	2	2.5	50
Continuous Paleness	10	13	50

**Table 4:** Anatomic characteristics of TOS patients found at surgery and the outcome.

Anatomic Characteristics	Number	%	Outcome Excellent or Good %
Scalenus muscle	*n* = 88		
normal	12	14	83
large	20	23	50
tendinous	38	43	71
tight posterior fascia	18	20	78
First rib-clavicle room	*n* = 78		
wide	58	74	69
somewhat narrowed	17	22	65
narrow	3	4	100
Estimated plexus compression	*n* = 79		
obvious	71	90	72
probable	8	10	63

**Table 5:** Long-term outcome after supraclavicular TOS surgery without first rib resection. Patients excluded (*n* = 5) due later rib resection and patients who did not respond the questionnaire contacted by phone (*n* = 47).

Assessment	Included 89 Patients Mean (SD) *	Failured 5 Patients Status after Rib Resection Mean (SD) *	By Phone Reviewed	*p*-Value
QuickDASH score (scale 0–100)				
At follow-up	37 (24)	49 (21)	-	0.279 **
CBSQ score (scale 0–120)				
At follow-up	52 (35)	59 (39)	-	0.665 **
Pain (NRS scale 0–10)				
Preoperatively	7.8 (1.9)	8.0 (1.2)	-	0.806 **
At follow-up	4.0 (3.2)	6.2 (1.9)	-	0.059 **
Numbness (NRS scale 0–10)				
Preoperatively	7.4 (2.0)	5.8 (3.6)	-	0.367 **
At follow-up	4.0 (3.0)	3.2 (3.1)	-	0.553 **
Weakness (NRS scale 0–10)				
Preoperatively	7.3 (2.0)	7.0 (1.7)	-	0.706 **
At follow-up	3.8 (2.9)	4.8 (1.9)	-	0.463 **
Daily harm caused by TOS (scale 0–10)				
Preoperatively	8.1 (1.6)	7.6 (0.9)	-	0.536 **
At follow-up	4.1 (3.1)	5.6 (2.6)	-	0.278 **
Results	Included 89 patients *n* (%)	Failured 5 patients Status after rib resection *n* (%)	By phone 47 patients *n* (%)	*p*-Value
Excellent or good Fair Poor	61 (74) 15 (18) 7 (8)	3 (60) 0 (0) 2 (40)	28 (60) 10 (21) 9 (19)	0.117

*CBSQ,* Cervical-Brachial Symptom Questionnaire; *QuickDASH*, Quick Disabilities of Arm, Shoulder and Hand; NRS, Number Rating Scale * = Data shown represent mean and its standard deviation, mean (SD). *p*-value ** = *t*-test comparison between groups.

## Data Availability

This study did not report any data.
